# Mechanical properties in crumple-formed paper derived materials subjected to compression

**DOI:** 10.1016/j.heliyon.2017.e00329

**Published:** 2017-06-18

**Authors:** D.A.H. Hanaor, E.A. Flores Johnson, S. Wang, S. Quach, K.N. Dela-Torre, Y. Gan, L. Shen

**Affiliations:** a Chair of Advanced Ceramic Materials, Technische Universität Berlin, 10623, Germany; b CONACYT − Unidad de Materiales, Centro de Investigación Científica de Yucatán, Calle 43, No. 130 Col. Chuburná de Hidalgo, Mérida, Yucatán 97205, Mexico; c School of Civil Engineering, University of Sydney, Sydney, NSW 2006, Australia

**Keywords:** Mechanical engineering, Materials science

## Abstract

The crumpling of precursor materials to form dense three dimensional geometries offers an attractive route towards the utilisation of minor-value waste materials. Crumple-forming results in a mesostructured system in which mechanical properties of the material are governed by complex cross-scale deformation mechanisms. Here we investigate the physical and mechanical properties of dense compacted structures fabricated by the confined uniaxial compression of a cellulose tissue to yield crumpled mesostructuring. A total of 25 specimens of various densities were tested under compression. Crumple formed specimens exhibited densities in the range 0.8–1.3 g cm^−3^, and showed high strength to weight characteristics, achieving ultimate compressive strength values of up to 200 MPa under both quasi-static and high strain rate loading conditions and deformation energy that compares well to engineering materials of similar density. The materials fabricated in this work and their mechanical attributes demonstrate the potential of crumple-forming approaches in the fabrication of novel energy-absorbing materials from low-cost precursors such as recycled paper. Stiffness and toughness of the materials exhibit density dependence suggesting this forming technique further allows controllable impact energy dissipation rates in dynamic applications.

## 1 Introduction

In recent decades mesostructural design has emerged as an important approach in materials science. With the term mesostructure we generally refer to a distinguishable structure within a bulk material that may exist at length scales higher than the material’s identifiable microstructure. For a given bulk material geometry and composition, by utilizing an interplay between mesostructure and microstructure one is able to achieve improved material properties that would otherwise be difficult to obtain. Mesostructured systems have most commonly been discussed in terms of tailored three dimensional structures in alloys and oxide systems [1, 2, 3], however such systems are by no means the only type of system in which the control of structure at an intermediate scale can be used to tailor material performance.

The fabrication of bulk material from flat or sheet-like precursors is frequently exploited with a view to imparting enhanced mechanical properties. Numerous artificial and natural materials are formed by the parallel layering of component materials to form stratified structures. Examples from nature include nacre, leaf-sheaths and bone structures [4, 5, 6]. The concept of stratified or layered structures finds widespread application in engineering materials such as toughened glass, fibre reinforced composites and armour [6, 7, 8, 9, 10]. In such materials mechanisms acting at layer interfaces serve to enhance the mechanical properties of bulk systems through the distribution of stress, disruption of crack propagation and accommodation of strain.

Further to layering, the disordered crumpling of a sheet like materials presents an alternative approach to the synthesis of a mesostructured bulk material from a 2D precursor. Recent years have seen growing interest in the morphological and mechanical aspects of structures formed by crumpling processes. Such systems exhibit structural complexity at intermediate lengthscales arising through self-avoidance and non-linear localised deformations. The mechanical attributes that consequently are imparted by such mesostructuring have been the subject of numerous emerging studies [11, 12, 13, 14, 15, 16]. The simple process of crumpling results in the formation of a highly complex network of folds and facets, and its topology cannot be described in a straightforward manner [17]. Sheet like materials in crumpled form are considered to be locally flat nearly everywhere, with the exception of a network of creases or ridges [18]. Through various mechanisms, these creases and surfaces deform under load and influence each other, potentially giving rise to high strength in the bulk. If we assume the thickness of a precursor sheet to be negligible relative to its other two dimensions, then a significant fraction of voids is expected in a crumpled ball even after a high level of volumetric compression [19].

Crumple-formed structures in terms of ridge networks, scaling behaviour and physical properties have been investigated to date using paper [20, 21], aluminum foil [12, 22, 23] and graphene [24] precursors. In such structures, morphological features exist across a range of length scales, and are reported to exhibit self-similar scaling, or fractality. For a given confining force, the relationship between the volume of a crumpled structure and the linear size of the precursor sheet is described by the systems fractal dimension D*_f_*, which describes the size and density of vertices in the structure [25, 26, 27] and has been shown to depend on the plasticity of the precursor material [14].

Most contemporary studies into morphologies arising from crumple forming have dealt with low density structures, such as those exhibited by crumpled paper balls [13, 18, 25]. In such systems an unusually high resistance to compression is found for systems of surprisingly low solids fraction, that is to say a crumple-formed material consisting primarily of empty space is found to exhibit high compressive strength not typically encountered for materials of such density [13]. However, crumple-formed materials of higher density, that is to say density comparable to that of typical engineering materials, are seldom investigated. An important recent study by Bai et al. [28], utilized a high pressure system to synthesize dense crumpled materials. These results suggest that crumple formed materials should exhibit good mechanical performance under conditions of high impact loading; however, experimental studies of such systems are not reported.

Crumple-forming represents an unexplored avenue towards the fabrication of low-cost engineering materials with good weight-specific mechanical attributes from diverse precursors including waste-material. This fabrication methodology offers new potential approaches towards materials recycling and can facilitate the more efficient utilisation of waste – streams, in particular waste paper and cardboard, allowing greater value-extraction and minimisation of landfill disposal. With this motivation, in the present work we report the first investigation of the mechanical properties of crumple formed materials. Here we examine the high pressure crumple-forming of bulk specimens from tissue paper precursor materials and study the compressive mechanical attributes achievable in these materials under conditions of quasi-static and impact loading.

## 2 Materials and methods

### 2.1 Specimen fabrication

**Fig. 1:**
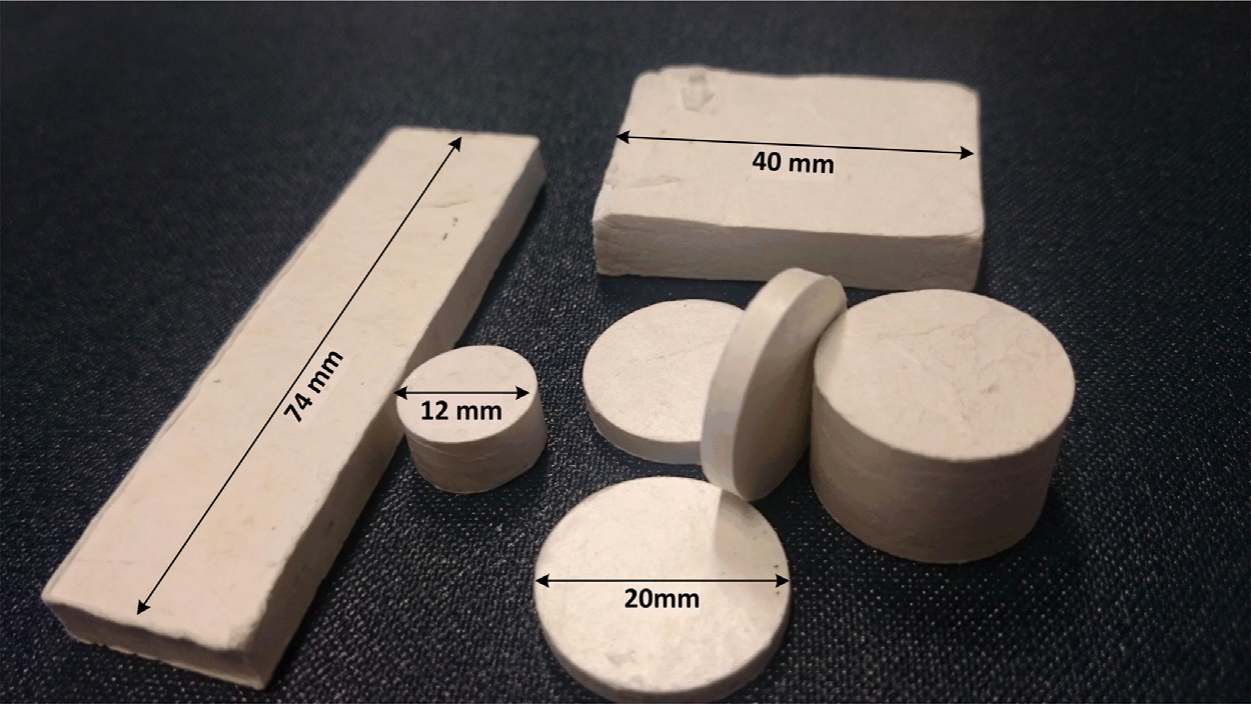
Crumple formed cellulose tissue paper specimens in a range of geometries.

It was found that crumple forming of rigid materials with densities comparable to engineering polymers (∼10 ± 0.5 g cm^−3^) can be achieved by hydraulic uniaxial confinement of randomly crumpled tissue paper, yielding specimens in a variety of sizes and geometries as illustrated in Fig. 1.

**Fig. 2:**
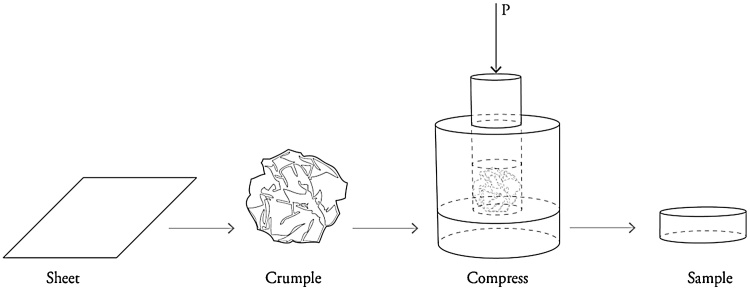
Schematic illustration of the crumple-forming process.

Cylindrical crumple-formed specimens used in the present work for quasi-static and high strain-rate compression characterization were manufactured from dust free tissue paper (Kimtech, Kimberly-Clark, USA), which consists of 100% virgin fibre cellulose with an average density of 0.02 kg m^−2^. Materials were fabricated at three different compression levels, with four specimens produced at each condition. For each specimen, a sheet measuring 150 × 300 mm was manually crumpled and packed into a steel die with an inner diameter of 12 mm. This dimension was chosen as it is compatible with both testing methodologies employed. Subsequently, uniaxial compression of the die was applied and maintained for durations of 20 seconds by means of a loading frame with applied pressure controlled in the regime 27–220 MPa to yield materials of varied density in the regime 0.86–1.34 g cm^−3^ as outlined in Table 1. A schematic illustration of the fabrication process is given in Fig. 2. The mass and dimensions of specimens were recorded subsequent to fabrication and these were then maintained in airtight containers prior to mechanical analysis in order to minimise material degradation as the result of humidity and/or temperature fluctuations. No measurable changes in specimen dimensions were observed between removal from the fabrication apparatus and mechanical testing, which was conducted in a matter of days subsequent to fabrication.

**Table 1:** Fabrication parameters for crumpled paper specimens. Four specimens were fabricated for each applied pressure.

Density classification	Specimen density range (g cm^−3^)	Applied pressure (MPa)	Mean thickness (mm)	Thickness range (mm)
Low	0.86–0.90	27	6.35	5.82–7.00
Medium	1.100–1.275	133	6.00	5.80–6.12
High	1.300–1.340	220	5.87	5.68–6.24

### 2.2 Mechanical characterisation

In order to understand the behaviour of crumple-formed structures under a range of stress conditions, rate effects must be considered in their characterization. Both quasi-static and high strain rate regimes were therefore implemented to determine the true stress-strain response of materials.

#### 2.2.1 Quasi-static testing

**Fig. 3:**
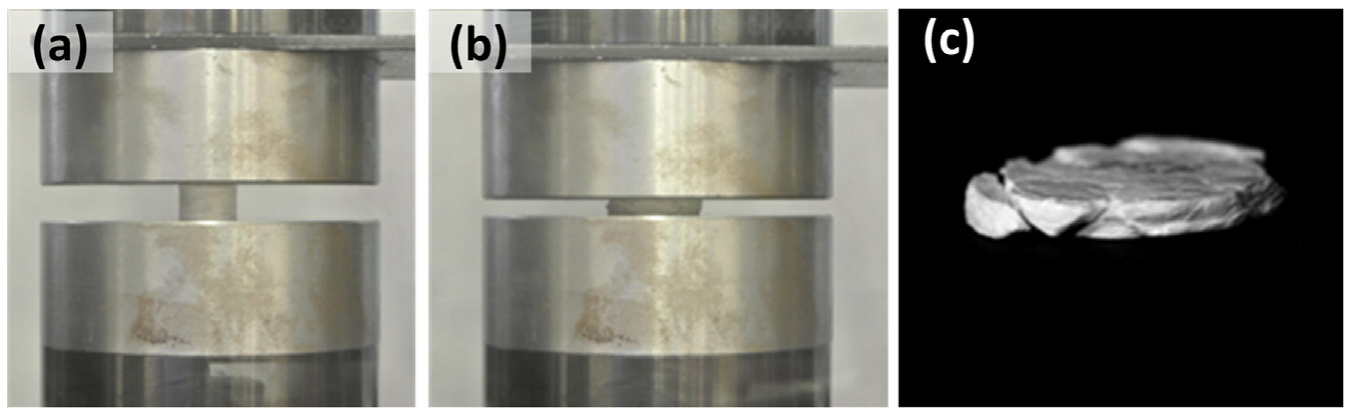
Typical quasi-static testing of 12 mm diameter specimens. Profile at (a) commencement and (b) termination of testing. (c) post compression specimen.

Quasi-static compressive testing was carried out using a MTS Criterion 43 loading frame with specimens positioned between two platens, which had been graphite-lubricated in order to minimise specimen barrelling, as shown in Fig. 3. A constant compression rate of 0.5 mm min^−1^ was maintained for all tests with vertical displacement monitored via a linear variable differential transducer (LVDT). Testing was undertaken up to a maximum compressive load of 40 kN. The acquired displacement and force data were used to determine values of Engineering Stress and Engineering Strain, defined as the nominal acquired values. Digital image analysis was used to capture the longitudinal and transverse deformation of the specimen at intervals of 2.5% strain and synchronize the specimen expansion profile with the recorded compressive load. This enables the extraction of true stress and true strain, defined respectively as the actual force applied over the actual contact area and the natural logarithm of the ratio of deformed size to original specimen height. A typical post failure specimen is depicted in Fig. 3(c). Four samples corresponding to each density range (low, medium and high) were considered giving rise to a total of 12 quasi-static tests. Owing to variations in specimen thicknesses, the effective applied strain rate varied from 0.00099 s^−1^ to 0.00144 s^−1^ with an average strain rate of 0.00130 s^−1^.

#### 2.2.2 High strain-rate testing

A split Hopkinson pressure bar (SHPB) was utilized for high strain-rate response characterisation of crumpled specimens. Both incident and transmission bars were composed of Aluminium 7075 alloy, with dimensions of 1500 mm in length and a diameter of 15 mm. A foam pulse shaper was further utilized and thus using a 300 mm long Al striker bar launched at a pressure of 7 bar, strain rates in the range 2800 to 4300 s^−1^ were achieved in the specimen. The incident, transmitted and reflected pulses were acquired through strain gauges placed on the incident and transmitted bars, allowing the determination of stress/strain data through Equations (1)–(3) [29]:

(1) $$\sigma_{s}\left(t\right)=\frac{A}{A_{s}}E\varepsilon_{t}\left(t\right),$$

(2) $$\varepsilon \left(t\right)=\frac{2C_{o}}{l_{s}}\overset{t}{\underset{0}{\int }}\varepsilon_{r}\left(t\right)dt\text{,}$$

(3) $$\overset{\prime }{\varepsilon_{s}}\left(t\right)=\frac{2C_{o}}{l_{s}}\varepsilon_{r}\left(t\right).$$

where ${ɛ}_{r}\left(t\right)$ and ${ɛ}_{t}\left(t\right)$ respectively denote the reflected and transmitted strains, Co is the bar wave velocity, ls is the initial specimen length, E is the modulus of elasticity of the bar material and A/As denotes the ratio of the bar area to the specimen area.

A total of 13 specimens of the three density classifications (Table 1) were tested by SHPB. In order to determine the true stress-strain behaviour, a high speed camera recording at 1 × 10^5^ frames per second was used to capture specimen deformation as a function of time, which was synchronized with the sample stress history.

## 3 Results

### 3.1 Quasi static compression

**Fig. 4:**
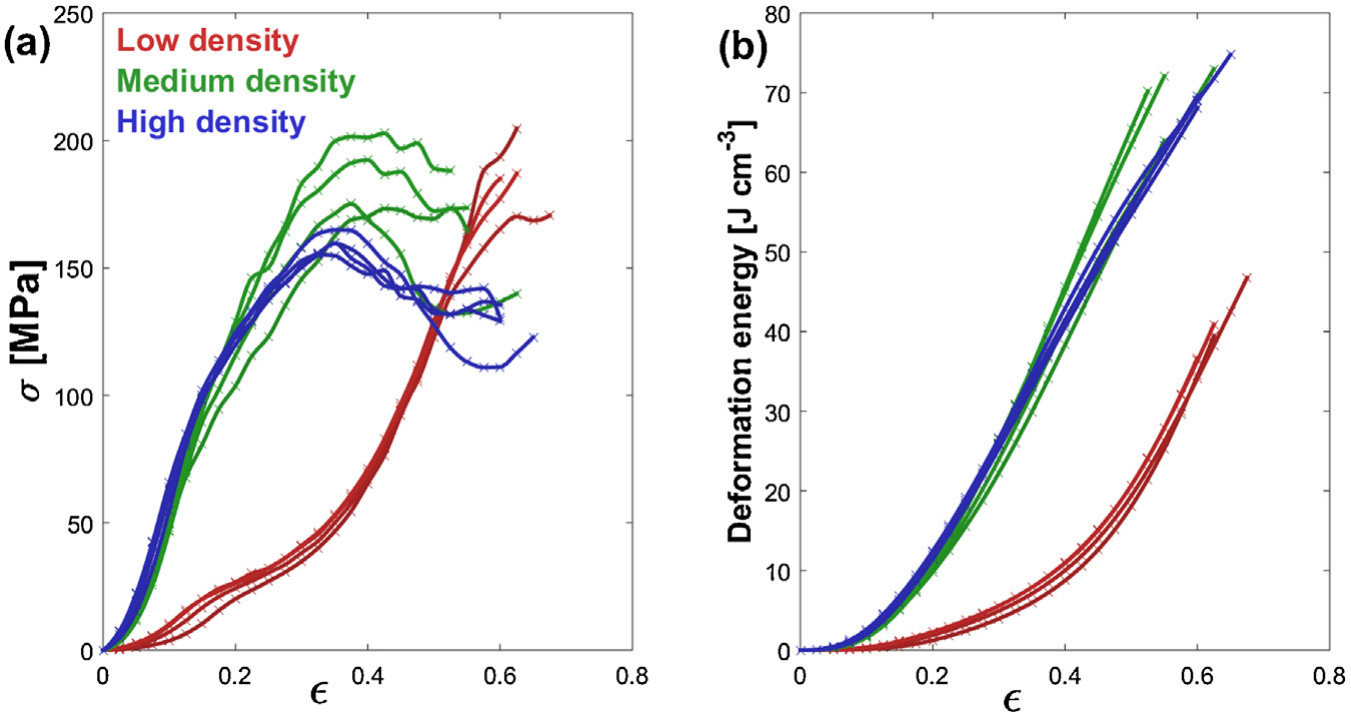
(a) True stress/strain behaviour of crumple formed materials of different densities under quasi-static compression (b) Deformation energy versus true strain.

Fig. 4 shows the true stress-true strain curves for crumple-formed specimens of different densities under quasi-static conditions. It can be seen that for the four high-density specimens maximum true stress levels in the range of 150–200 MPa (with an average of 172 MPa) are achieved prior to specimen failure at around 0.35 strain. Fig. 3(c) shows a yielded specimen, exhibiting shear type failure indicated by inclined fracture lines. Two distinctive regimes are observed in high-density specimens before peak stress, which are a pseudo-elastic regime up to a strain of ∼0.15 with a stress of around 100 MPa and a pseudo-plastic regime up to failure strain. This behaviour can be understood if we consider crumple-formed specimens as a cellular material that densifies with compressive strain leading to greater frictional energy dissipation friction arising from an increasing contact area. A further contribution to the load resistance is expected also from ridge formation with associated plastic deformation [14]. For the four specimens of low density materials, the behaviour is similar to that of a typical foam in which crushing is the main mechanism of energy absorption; however, the contribution of friction and the creation of ridges results in a hardening-like regime up to ∼0.3 strain. Following this, a densification regime is identified where the stress increases rapidly with further increase of strain. Specimens of lower density do not exhibit distinguishable yield points, rather deforming plastically as voids are closed and overall hardness increases gradually. This results in different energy dissipation levels for a given strain as shown in Fig. 4(b). The behaviour of these materials is advantageous towards impact absorption applications as the density of crumpled structures, and the topology of voids and ridges can be tailored to yield energy dissipation at different targeted rates [30].

### 3.2 High strain-rate compression

**Fig. 5:**
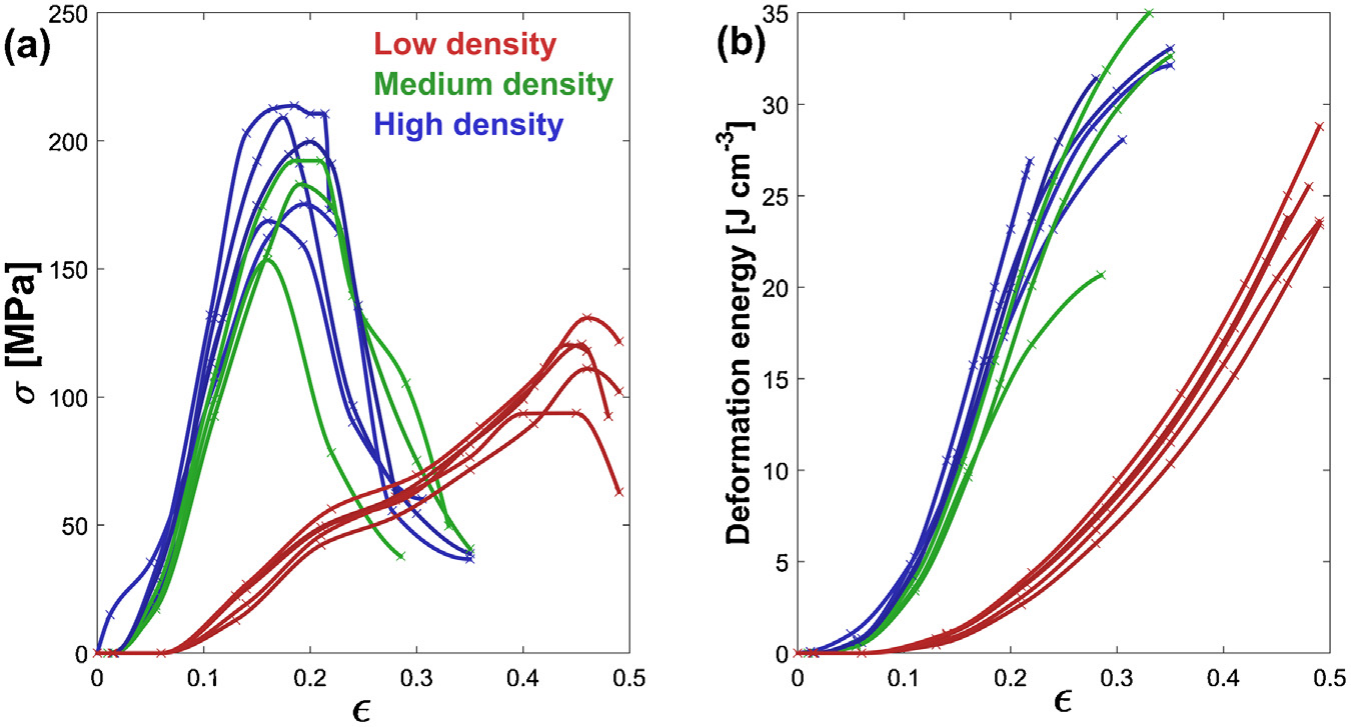
(a) True stress/strain behaviour of crumple formed materials of different densities in high strain rate compression (b) Deformation energy versus true strain.

**Fig. 6:**
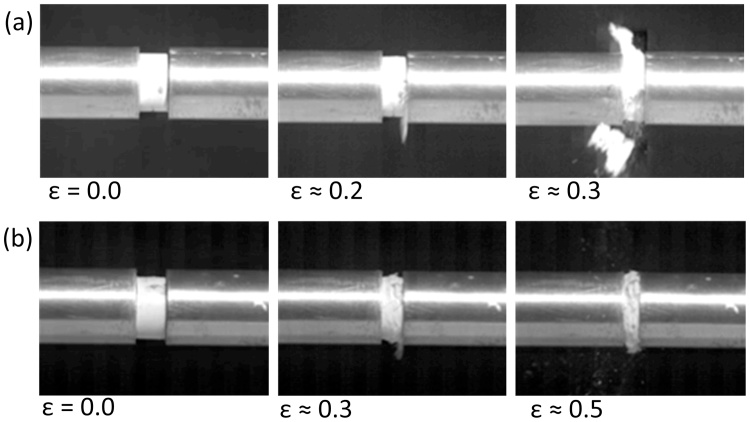
High speed imaging frames showing high strain-rate loading of 12 mm diameter crumple-formed material: (a) high-density specimen, (b) low-density specimen.

True stress-strain data from the high compressive strain-rate characterisation of specimens using a split Hopkinson pressure bar is shown in Fig. 5. For the four high density specimens, it can be seen that true maximum stress levels are in the range of 150–210 MPa (with an average of 191 MPa) which is somewhat higher than the stress levels observed in quasi static compressive tests; however, failure through specimen rupture occurs at ∼0.2 strain as shown in the sequence of frames from high speed imaging in Fig. 6. This behaviour indicates that at high strain rates the high density crumpled material becomes stiffer, absorbing energy at a higher rate than in the quasi-static case; however, the material also becomes brittle and fails at lower strain levels. For low density specimens, the behaviour is similar to that observed in quasi-static loading conditions, although still exhibiting a higher energy absorption rate. The strain-rate effect on stiffness may be due to entrapped air as in foams [31] or due to the strain rate dependence of cellulose [32].

### 3.3 Density effects

**Fig. 7:**
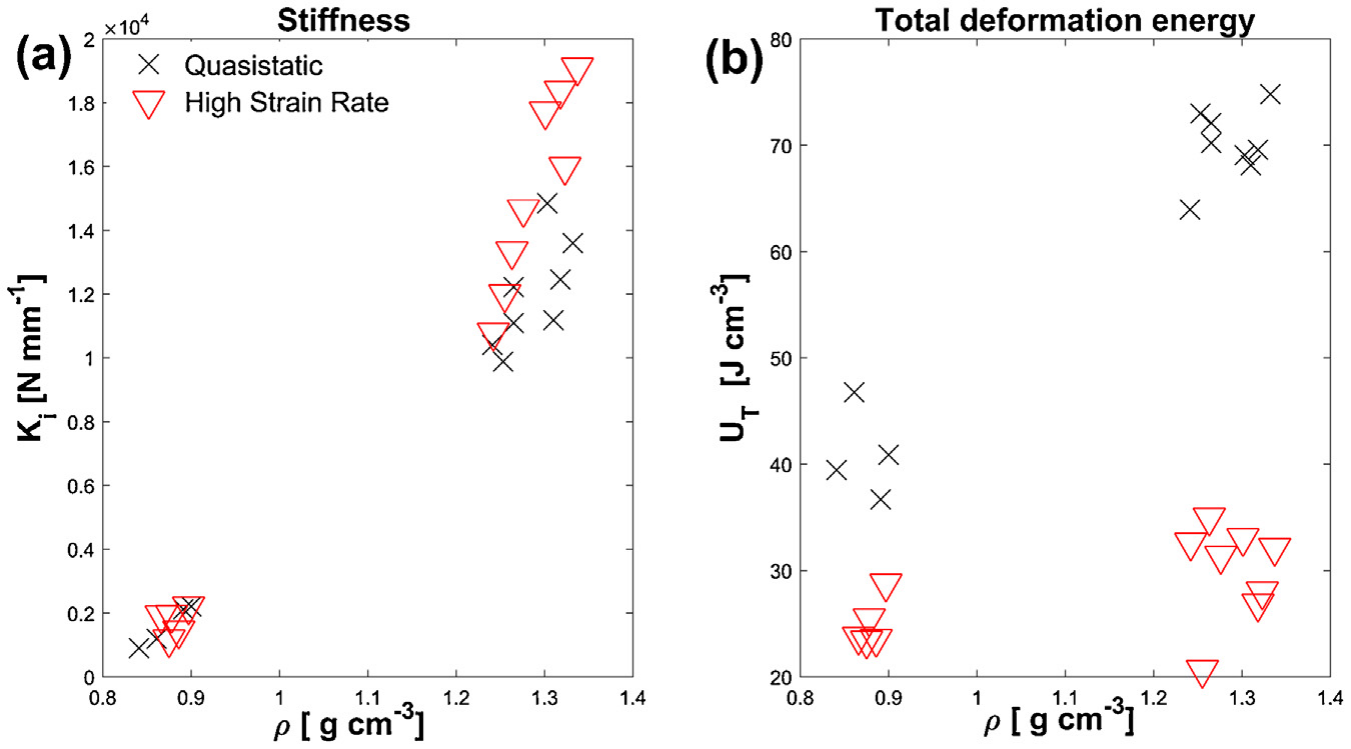
Variation with density of (a) initial stiffness and (b) total deformation energy.

Fig. 7 illustrates the significance of density on the mechanical behaviour exhibited by materials in the present work. In general, materials of lower density exhibit more gradual energy dissipation and lower initial stiffness, K_i_, given as the stiffness up to 10% strain. Total deformation energy U_T_, a parameter of importance in materials for impact absorbing applications, is higher in quasistatic conditions, as can be seen in Fig. 7(b). This parameter is defined as the volume specific energy dissipated during compression, calculated from an integration of the true stress/strain data. In contrast to quasistatic conditions, materials of higher density do not absorb significantly more energy per unit volume under high strain rate conditions representative of shock impact.

## 4 Discussion

The mechanics of crumpled systems have been examined in earlier studies, suggesting a porous structure comprising fractal networks of ridges and facets may impart high compressive strength through localised deformation. However, these investigations were not conducted with a view towards materials processing or recycling applications. The present work constitutes the first time that this approach to mesostructuring has been applied to the fabrication of higher density materials through high pressure confinement, resulting in materials with densities comparable to many engineering materials. Crumpling processes were applied using uniaxial compression in the present work, however isostatic compression processing techniques (Cold Isostatic Pressing, CIP) would similarly be applicable for the implementation of such crumple-forming. The range of pressures applied here is well within the range attainable by industrial CIP fabrication systems [33], suggesting similar materials could be produced on a large scale in a diverse range of geometries using waste material feedstock. Naturally, the actual applied pressure required for crumple-fabrication in CIP methods would be contingent on precursor material yield characteristics and the product geometry.

Medium and high density crumple formed materials were found here to have ultimate compressive stress values in the approximate regime 150–210 MPa under loading in both quasi static and high strain rate loading conditions. In these experiments, the true stress was calculated on the basis of the actual true cross-sectional area (Section 2.2) and is considered to be homogenous. It should be noted that at high strains, specimens exhibited some barrelling (See Figs. 3 (b) and 6 (b)) owing to friction between specimen and jigs, introducing some heterogeneity in the pseudo-cellular materials’ stress state, resulting in a particular deformation and complex stress inside the specimen subjected to large compressions.

**Fig. 8:**
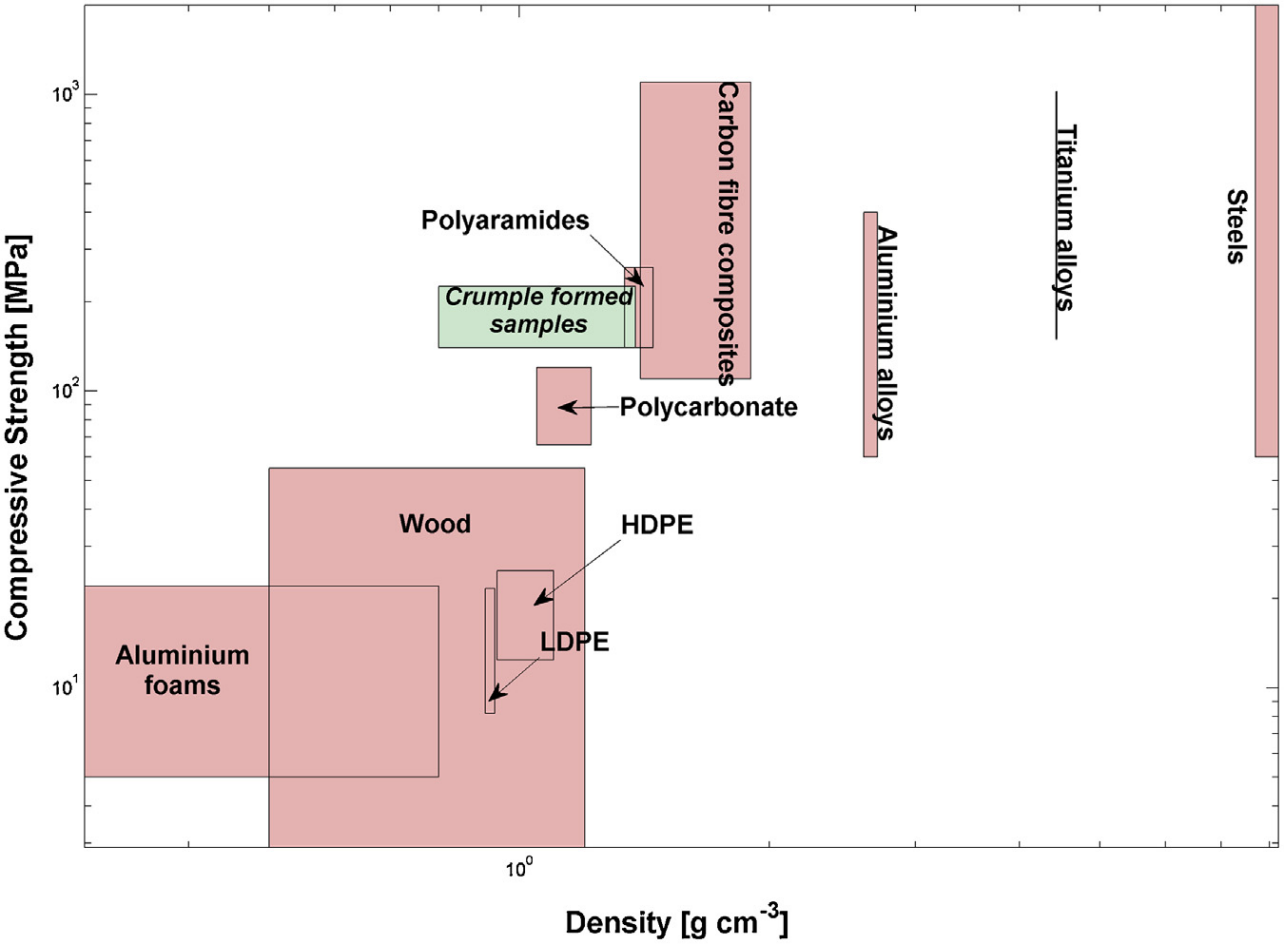
Materials performance comparison map in terms of compressive strength and density. Boxes indicate approximate range of properties.

The Ashby map of Fig. 8 shows that the compressive stress values, taken from specimens exhibiting distinct failure, compare favourably with reviewed of performance data for engineering materials considering parameters of density and compressive strength [34, 35, 36, 37, 38, 39, 40, 41, 42]. While certain polyaramides and carbon fibre reinforced polymers do exhibit superior compressive performance at only slightly higher densities, the high cost of these materials is a clear disadvantage relative to the materials tested in the present work, which can readily be fabricated from tissue paper, and potentially from pulp or recycled materials.

The crumple-formed materials fabricated in the present work were prepared using a simple cellulose tissue paper precursor, a material of minor engineering significance. At the microscale this type of material does not exhibit particularly advantageous mechanical properties and exhibits similar mechanical behaviour to wood [43] or short-chain polymeric compounds [42, 44]. Through mesoscale structuring, achieved by confinement at high pressure, these materials showed surprisingly high levels of energy dissipation coupled with high levels of strain to failure in the regime 0.2–0.4, with deformation energies in the range 20–35 J cm^−3^ and 35–75 J cm^−3^ for high strain rate and quasistatic conditions respectively under the given testing conditions applied. Moreover, the rate of impact dissipation, evident in the steepness of the deformation energy curve under conditions of high strain rate loading, demonstrates a dependence on the materials density. This suggests that similar materials can be designed to impart tailorable dissipation rates in impact protection and vibration damping applications where the moderation of deceleration rates is of great importance [30].

It is likely the energy dissipation seen in these crumple formed materials occurs through frictional mechanisms distributed across many scales and orientations within the 3-D structure. The mechanical properties found in the present work further suggest that crumple forming is a feasible and versatile approach towards fabricating low-cost engineering materials from waste material feedstock in a broad range of macroscopic geometries. The materials fabricated in the present work further demonstrate the potential to utilize sheet-like precursors in a crumple-forming process as a means towards the recycling of waste materials towards the fabrication of rigid materials with enhanced mechanical properties.

## 5 Conclusions

Crumple formed materials were fabricated by the uniaxial compression of a cellulose tissue precursor. Fabricated specimens demonstrate the efficacy of this forming technique for the production of rigid materials from low-cost sheet-form precursors. The materials’ mechanical response was studied in quasi static and high strain-rate compression conditions. True stress/strain behaviour was found to compare favourably with engineering materials of similar density suggesting crumple-forming is an attractive route to the fabrication of acceptably strong structural materials from low-cost and/or recycled input materials.

High strain rate behaviour indicates that controlling the density of crumple-formed materials by varying the applied pressure in fabrication, can serve as an effective route to moderate impact dissipation rates, offering a new approach to tailorable deceleration in protective systems.

The findings of the work presented here highlight the attractiveness of crumple-forming in materials processing and recycling and provide an impetus to the furthering of our understanding of similar materials in terms of forming methods, precursor materials and mechanistic aspects of energy dissipation.

## Declarations

### Author contribution statement

D. Hanaor: Conceived and designed the experiments; Performed the experiments; Analyzed and interpreted the data; Contributed reagents, materials, analysis tools or data; Wrote the paper.

E. F.-Johnson: Conceived and designed the experiments; Performed the experiments; Analyzed and interpreted the data; Wrote the paper.

S. Wang, S. Quach and K. D.-Torre: Performed the experiments; Analyzed and interpreted the data.

Y. Gan and L. Shen: Conceived and designed the experiments; Analyzed and interpreted the data; Wrote the paper.

### Competing interest statement

The authors declare no conflict of interest.

### Funding statement

This research did not receive any specific grant from funding agencies in the public, commercial, or not-for-profit sectors.

### Additional information

No additional information is available for this paper.
